# Illinois State Medical Society Circular

**Published:** 1857-09

**Authors:** 


DEAR SIR:—At the Annual Meeting of the Illinois State Medical Society, held at Chicago on the first Tuesday in June last, the undersigned were appointed a Committee to report on Practical Medicine.

In order to fully accomplish the object of the appointment, answers to the following questions are respectfully requested:

1. What have been the most prevailing diseases in your locality during the year? Give causes, symptoms, treatment and rate of mortality.

2. Has any unusual epidemic prevailed? If so, wherein have the symptoms differed from those seen in the same disease when occurring sporadically?

3. Is Typhoid Fever more prevalent than in former years? Does genuine Typhoid Fever prevail on the prairie? If so, does it present the same appearances as when found in the *timber?* Give distinctions, if any.

4. Do the ordinary diseases of your region seem to be undergoing changes from year to year, requiring different treatment? If so, what are those changes, and what different treatment is necessary?

5. What improvements have you to suggest in the treatment of any disease? Any new remedies to recommend, or any new applications of old remedies?

6. Has *Stomatitis Materni* prevailed in your locality? Have you ever met with a similar form of disease in infants or in men? Does the disease prevail most in miasmatic districts? Give suggestions as to causes and treatment.

7. Does *Milk Sickness* prevail in your vicinity? Is it Animal, Vegetable or Mineral in its origin?

8. Has *Cholera Infantum* prevailed with you during the year? Is it produced or modified by miasmatic causes. Do you agree with the opinion expressed by writers in the "Books?"

9. Please give topographical description of your locality, together with any facts that may come under your notice which will be interesting to the medical profession.

Please address F. K. Bailey, M. D., Chairman of Committee, at Joliet, Will Co., Ill., as soon as 1st April, 1858.

F. K. Bailey,<br>

D. W. Stormant, } Committee.<br>

A. J. Crain,

